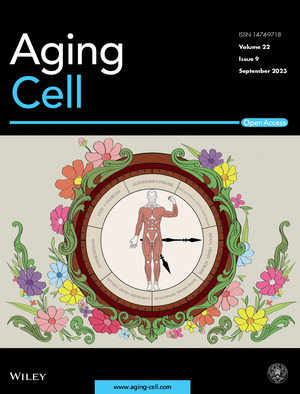# Additional Cover

**DOI:** 10.1111/acel.13993

**Published:** 2023-09-12

**Authors:** Chaojie Ye, Lijie Kong, Yiying Wang, Jie Zheng, Min Xu, Yu Xu, Mian Li, Zhiyun Zhao, Jieli Lu, Yuhong Chen, Weiqing Wang, Guang Ning, Yufang Bi, Tiange Wang

## Abstract

Cover legend: The cover image is based on the Research Article *Causal associations of sarcopenia‐related traits with cardiometabolic disease and Alzheimer's disease and the mediating role of insulin resistance: A Mendelian randomization study* by Chaojie Ye et al., https://doi.org/10.1111/acel.13923